# Comment on: “Comparison of endoscopic submucosal dissection and transanal endoscopic surgery for the treatment of rectal neoplasia: A systematic review and meta-analysis”

**DOI:** 10.1016/j.clinsp.2025.100663

**Published:** 2025-04-24

**Authors:** Wensi Ouyang, Guimei Guo

**Affiliations:** Changchun University of Chinese Medicine, Changchun, Jilin, PR China

*Dear Editor,*

We read with great interest the systematic review and meta-analysis by de Sousa et al. titled “Comparison of endoscopic submucosal dissection and transanal endoscopic surgery for the treatment of rectal neoplasia: a systematic review and meta-analysis”.^1^ This study provides valuable insights into the comparative efficacy and safety of Endoscopic Submucosal Dissection and Transanal Endoscopic Surgery for early-stage rectal tumors, offering guidance for clinical decision-making. However, I would like to provide several points for consideration to strengthen the interpretation and applicability of the findings.

Firstly, the authors conducted a systematic search across MEDLINE, EMBASE, Cochrane, and LILACS databases. However, several other well-known databases, like PubMed, Web of Science, and China National Knowledge Infrastructure, are also of great significance. Expanding the search scope to incorporate these databases could enhance the comprehensiveness of evidence synthesis, reduce selection bias, and improve the robustness of the meta-analytic conclusions.

Secondly, the meta-analysis included several double-zero-event studies in the pooled estimates of outcomes such as en bloc resection rate, R0 resection rate, perforation rate, bleeding rate, procedure time, and hospital stay. The inclusion of these studies may introduce bias, as the absence of effective data in these studies can distort the overall effect estimates and confidence intervals. To enhance the accuracy and reliability of the meta-analysis results, it is advisable to exclude double-zero-event studies.

Finally, while the authors’ adherence to PRISMA guidelines is commendable, their omission of sensitivity analysis and publication bias assessment in this meta-analysis. This oversight may compromise the reliability of the result interpretation. It risks misguiding the understanding of overall effect estimates, and clinical decisions and impacting policymaking, and ultimately undermining the scientific credibility and transparency of the study.

In conclusion, we commend de Sousa and colleagues for their significant contributions in this field. If the authors can address these issues, it will further improve the rigor and reliability of their research findings. It would also be able to advance the evidence-based practice of minimally invasive interventions for early-stage rectal tumors.

## Ethical approval

Not applicable.

## Funding

No funding was received for this research.

## Conflicts of interest

The authors declare no conflicts of interests.
